# Prevalence and Associated Factors of Depressive Disorder among Prisoners in Mekelle General Prison Center, Tigray, Ethiopia: A Cross-Sectional Study Design

**DOI:** 10.1155/2021/1942674

**Published:** 2021-06-01

**Authors:** Solomon Gidey Welu, Desta Hailu Aregawi, Hagos Tsegabrhan Gebreslassie, Kokob Gebru Kidanu

**Affiliations:** ^1^Department of Psychiatry, College of Health Science, Mekelle University, Mekelle, Ethiopia; ^2^Department of Maternal and Reproductive Health Nursing, College of Health Science, Mekelle University, Mekelle, Ethiopia

## Abstract

**Background:**

Depression is the most prevalent mental disorder among prisoners and is the second leading cause of disability worldwide. Depression affects more for those who are less educated, female, single, and young prisoners, and worldwide prevalence of depression among prisoners is 10.2% and 14% for male and female prisoners, respectively. However, a study conducted on prevalence of depression and associated factors is scarce in Ethiopia (Tigray) despite there is high magnitude. *Methodology*. An institution-based cross-sectional study was conducted among randomly selected 414 prisoners in Mekelle General Prison Center. Data were collected from April to May 2019. A structured and standardized data collection tool (PHQ-9) was used. Bivariate and multivariable logistic regression analysis was carried out by SPSS version 20. Statistical significance was determined at *P* value < 0.05.

**Result:**

In this study, 408 prisoners had participated. The prevalence of depression among prisoners was found to be 228 (55.9%; 95% CI: 51.2%, 61%). Being unemployed and student, lifetime substance use, history of child abuse, weight loss in prison, quality of meal in prison, being not happy inside prison, being sentenced for more than six years, and poor and moderate social support were significantly associated with depression.

**Conclusion:**

Prevalence of depression among prisoners was found to be high (55.9%). Prisoners who had lifetime substance use, being unemployed and student, history of childhood abuse, weight loss inside prison, being sentenced for more than six years, not happy inside prison, lack of social support, and poor quality of prison meal were more likely to have depression. Thus, giving training to strengthen social support, giving training on how to cope up with prison environment, giving training to scale up a happy life, and improving quality of prison meal as well as mental health service will help to reduce the problem. Conducting interventional study is relevant.

## 1. Background

Worldwide, around 10 million people are found in prisons and most of them are from low- and medium-income countries (LMICs) [1–4]. Ethiopia has 93,044 prisoners and it has the highest number of prisoners next to South Africa which has 165,395 in Africa [5]. Although mental disorder affects all the community significantly (schizophrenia (0.3-0.7%) and depression (4.4%)), there is high prevalence of mental disorder among prisoners (schizophrenia (4%), depression (10%, male and 14%, female)) than the general population [3, 6–12]. Over the entire world, there are 450 million people who suffered mental disorder, but there is insufficient mental health service [13]. Worldwide, one in four people is affected by mental disorder [14]. The mental health treatment among the LMICs is very low and they have large treatment gap [15]. Mental disorders cause significant burden across the world, which accounts 13% of global burden of disease. Most of them, i.e., 80% of people with psychiatric disorder, are in LMICs. Psychiatric disorders are the major cause of disease-related disability; particularly, depression is the 2^nd^ leading cause of disability [16]. No health without mental health [15, 17]. As public health institute estimates that one-third of population meet the criteria for at least one mental disorder in the 12-month prevalence and around half of the population suffer from mental disorder in their lifetime prevalence [8]. In Ethiopia, burden of mental illness comprised 11% of the total burden of disease, with schizophrenia and depression (3^rd^ leading cause of burden of diseases) included in the top ten most burdensome conditions, out-ranking [18, 19]. The prevalence of depression, which is one of the common mental disorders in Ethiopia, is 20.5% [19], but still it is underdiagnosed and undetected disorder which causes disability. Mentally ill patients and their family experience stigma, discrimination, and human right abuse in their daily life [18]. The report from the World Health Organization (WHO) in 2017 indicated that 322 million people live with depression worldwide (4.4%) [7]. Also, systematic review of 83 studies reported that prevalence of depression among outpatients was 27% [20]. A survey from 12 countries indicates that prevalence of depression among prisoners is 10% and 12% among men and women, respectively [21]. Prison population increases by one million per decade worldwide, and the worldwide estimated prevalence of depression among prisoners is 10.2% and 14.1% for male and female prisoners, respectively [22]. Many studies reported that prevalence of depression among prisoners were 42%, 43%, 81%, 85%, 35.3% and 37% in Iran, New York, India, Pakistan, Nepal, and Nigeria, respectively [23–28]). In three regions of Ethiopia, the prevalence of depression among prisoners is 43.8%, 56.4% and 41.9% in northwest Amhara, southern Ethiopia, and Jimma, respectively [11, 29, 30].

The most serious impact of depression is suicide [31, 32] which is the main cause of death in prison; a study done in Australia indicates that one-third of inmates reported lifetime suicidal ideation and one-fifth had attempted suicide [33]. Depression among prisoners causes a great disability which is 18%; of these, female prisoners are considered to have greater proportion of disability (55%) than males (34%) [34]. Also, depression causes premature morbidity and mortality, dysfunction of occupational and personal life due to loosing of working days, stigma, and economic crisis due to unemployment and cost for treatment [19, 35, 36]. Depression is high among those who are less educated, single, female, and young people [7, 33]. The consequence of untreated depression in prison ranges from severe deterioration of health and disability to suicide [31].

There were many factors in prisons that have contribution on mental health, particularly on depression including age, marital status, gender, substance abuse, overcrowding, family history of mental illness, feeling that life after discharged from prison will be difficult, history of chronic illness, history of childhood abuse, duration of imprisonment, suicidal ideation and plan to commit suicide, poor social support, unemployment, inadequate mental health service and recreational activities in prison, low quality of meal, previous incarceration, long stay at prison, having no visitors, no privacy, isolation, past psychiatric history, and bad memory about the crime [11, 26, 28–31, 37]. While most of prisoners are found at their productive age and expected to contribute to their society after return from their charges, the attention given to the most prevalent cause of disability (mental disorder) is very low [38]. Many prison studies indicated that the prevalence of mental illness is high, which indicates prison populations are vulnerable to mental illness. From the morbidity of mental illness, depression and anxiety are the highest and become the burdened disease at prison [31, 39].

However, the emphasis given to mental health was very low across the world in general particularly for prisoners. There is limited information about the magnitude of depression among prisoners who were incarcerated in Ethiopia particularly in Tigray, and generally, there is lack of information about prisoner's health condition. Even though health care service for mental disorder was designed in the national health policy of Ethiopia, interventions against the problem were very limited, which might be due to limited information about the problem. Thus, establishing the prevalence rates of mental disorders, particularly depression, has great importance in improving mental health services in prison. As a result, this study is aimed at assessing magnitude of depression and identifying its associated factors among prisoners detained in Mekelle General Prison Center, Ethiopia, which will serve as an input for policy makers, health service planners, and strategy designers.

## 2. Methods

### 2.1. Study Design

An institution-based cross-sectional study was conducted to determine the magnitude of depression and associated factors among prisoners found in Mekelle General Prison Center, Tigray, Ethiopia, from April to May 2019.

### 2.2. Study Setting and Data Collection Procedure

Mekelle is the capital city and the political and economic center of the Tigray Region, which is located in the northern part of Ethiopia and away by 783 kilometers from Addis Ababa, the capital city of Ethiopia. Mekelle City has seven subcities, and the total population of Mekelle City is estimated to be 406,338 [40]. Mekelle City has four health centers, three general hospitals, and one comprehensive specialized referral hospital. Mental health service is available in both the general hospitals and comprehensive specialized hospital. Mekelle has also one general prison center which is located in the western part of Mekelle City with varying numbers of prisoner population of each month between the range of 1300 and 1600 and the quarter year report prisoners of 1487. Also, it has temporary jails in each subcity. Mekelle General Prison Center has one clinic (has no psychiatric professional), school (elementary, high school, and college), cafeteria, one debit bank, and 9 buildings for prisoners. Also, it has religious rooms for praying.

Interviewer administered a structured and standardized questionnaire which was used to collect the data. The questionnaire had five parts: (1) sociodemographic data, (2) prison-related questions, (3) health-related questions, (4) Oslo Social Support Scale (OSSS) to assess level of social support, and (5) Patient Health Questionnaire (PHQ-9) scales to assess the depression and its associated factor. Data was collected by six BSc psychiatry professionals who work at Ayder Comprehensive Specialized Hospital (ACSH). Two MSc students were recruited as supervisors. The principal investigator and the supervisors checked the filled questionnaires for completeness and consistency. PHQ-9 is a tool which contained nine questions; each question measures a problem that the prisoners were bothered in the last 15 days was used to measure depression with scale measurement ranging from zero (not at all) to three (nearly every day). The data abstraction format was pretested in 5% of total sample size in Wukro General Prison Center two weeks prior to the main data collection time. The final tool was developed with some modifications after a thorough and deep review of inputs obtained during the pretest. The pretested participants did not participate in the study. Regular supervision was carried out by the supervisor and principal investigators during data collection. Each day during data collection, the filled questionnaires were checked for completeness and consistency. Data collectors had searched for three days to the participants who were absent during data collection. Data collectors and the supervisors were trained for one day by the principal investigator on the study/instrument, on the consent form, on how to maintain confidentiality, and on the data collection procedure.

### 2.3. Study Participants

#### 2.3.1. Eligibility Criteria

Prisoners who were 18 years and above and who can speak Tigrigna and Amharic were included in the study, while prisoners who were unable to communicate, awaiting for the trial, and imprisoned for less than 2 weeks were excluded. All prisoners found in Mekelle General Prison Center were the source of the population, and prisoners who were found at Mekelle General Prison Center during the study period were the study population. A simple random sampling technique was used to select 414 participants. Participants were selected randomly by a lottery method from study population frame.

### 2.4. Variables

#### 2.4.1. Dependent Variables

- Depression

#### 2.4.2. Independent Variables

- Sociodemographic variables—age, sex, religion, educational status, occupation, marital status, and income
- Psychosocial factors—social support, substance use, and history of childhood sexual abuse
- Health-related variables—history of previous mental illness, family history, chronic medical condition, suicide, loss of weight, and a frequent follow-up with a health care provider
- Prison-related variables—type of prison, thinking life will be complicated after release, not working inside prison, previous incarceration, satisfaction of life before imprisonment, short prison stay, recreational activities, and bullying

OSSS is a tool used to assess the level of social support with a score of 3-8 for poor support, 9-11 for moderate support, and 12-14 for strong support [41]. Based on PHQ-9, prisoners who score ≥5 were considered as having depression and prisoners who score less than 5 were considered as free of depression. Total score and depression severity is determined as follows: 1-4 for minimal depression, 5-9 for mild depression, 10-14 for moderate depression, 15-19 for moderately severe depression, and 20-27 for severe depression [42].

### 2.5. Sample Size

The sample size (*n*) was calculated by a single population proportion formula [*n* = [(*Z*_a/2_)^2^∗*P* (1 − *P*)]/*d*^2^] by assuming 95% confidence level, 5% margin of error (*d*), and 43.8% proportion (*P*) from northwest Amhara prisoners [29]. Based on the calculation, the total sample size was 414 prisoners.

### 2.6. Data Analysis and Processing

Data was coded, entered, edited, and cleaned using EpiData version 4.4.2.1. Data was exported to Statistical Package for Social Sciences (SPSS) version 20. Then, the data was analyzed to generate descriptive statistics: means, frequency, percentages, and standard deviations, using SPSS version 20. Data was presented using narrative, figure, and table form from the result of frequencies and crosstabs. Binary logistic regression analysis was used to calculate adjusted odds ratios to control for confounding variables with 95% confidence interval. All independent variables were entered to bivariate logistic regression, and factors result *P* value of less than 0.2 in bivariate were entered to multivariable logistic regression to detect the association. Statistical significance was determined at *P* value of 0.05.

## 3. Result

### 3.1. Participants

From 414 total sample sizes, four hundred eight participants were interviewed making a response rate of 98.5%. The reason for nonresponse was six selected participants who refused to disclose their information.

### 3.2. Prisoner's Sociodemographic Characteristics

The mean age of participants was 29 years (standard deviation, ±12.2), and majority of participants were males (396) (97.1%). Two hundred twenty-six (55.4%) participants were single, and majority of participants were Orthodox Christian followers (393) (96.3%). The mean income of participants before imprisonment was 3578.6 Ethiopian birr. About half of prisoners (211) (51.7%) were doing in their private work and one hundred seventy-one prisoners (41.9%) had educated in the elementary school (Table 1).

### 3.3. Psychosocial-Related Characteristics of Prisoners

**Figure 1:**
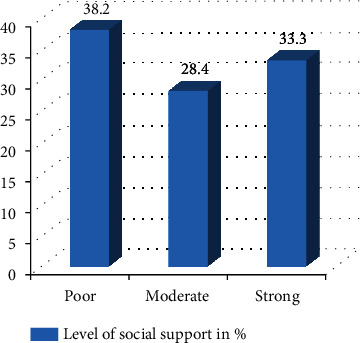
Level of social support among prisoners in Mekelle General Prison Center, Tigray, Ethiopia, 2019 (*n* = 408).

**Figure 2:**
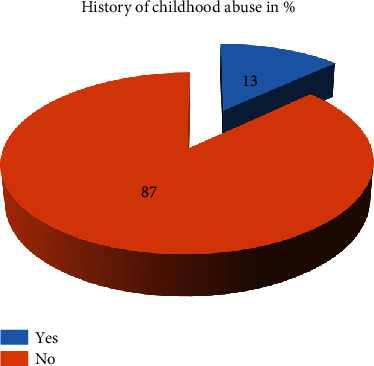
History of childhood abuse among prisoners in Mekelle General Prison Center, Tigray, Ethiopia, 2019 (*n* = 408).

Above one-third prisoners had poor social support (156) (38.2%), and one in every ten prisoners had history of childhood abuse (Figures 1 and 2).

### 3.4. Clinical Characteristics of Prisoners

From total participants, 21 (5.1%) of the prisoners had history of mental illness and fifty-nine (14.5%) of participants had history of chronic physical illness. About 35 (8.6%) of participants had family history of mental illness. Around one-fifth of participants subjectively reported that they have poor health status (78) (19.1) and about sixty-nine (16.9%) had a frequent follow-up with a health care provider. More than half of study participants reported that they have decreased their weight inside prison (229) (56.1%) (Table 2).

### 3.5. Prison-Related Characteristics of Prisoners

About 48 (11.8%) of study participants were previously incarcerated, and the most reason for imprisonment was theft or robbery (151) (37%). Around half of study participants (200) (49%) were sentenced for the range of 1-3 years and sixteen (3.9%) were charged for lifetime. The mean length of participants stayed in prison was 20.99 months. Two hundred thirty-six (57.8%) of study participants accepted their crime, and majority of the participants (290) (71.1%) had work inside prison. Majority of study participants (386) (94.6%) had satisfaction with day-to-day activity before imprisonment, and most of participants were participated in religious activity. Out of total participants, about three-fourth (308) (75.5%) were subjectively reported that quality of meal in prison cafe was bad. One-third of study participants were happy in prison, while majority of participants were not happy and only 14% of participants were faced bullying inside prison by police or prisoners (Table 3).

### 3.6. Substance and Suicide Characteristics of Prisoners

**Figure 3:**
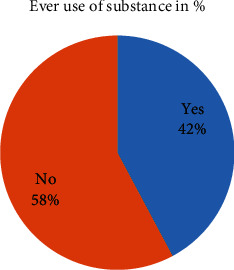
Ever use of substance among prisoners in Mekelle General Prison Center, Tigray, Ethiopia, 2019 (*n* = 408).

Out of total study respondents, 172 (42.2%) used substance in their lifetime. From these, 62.2% used only alcohol and 25.6% used alcohol, khat, and cigarette. About one in every ten study participants had suicidal ideation (59) (14.5%), and minority of study participants had history of suicidal attempt (27) (6.6%). The most frequent method to attempt suicide was hanging (29.6%) (Figure 3 and Table 4).

### 3.7. Prevalence of Depression among Prisoners

**Figure 4:**
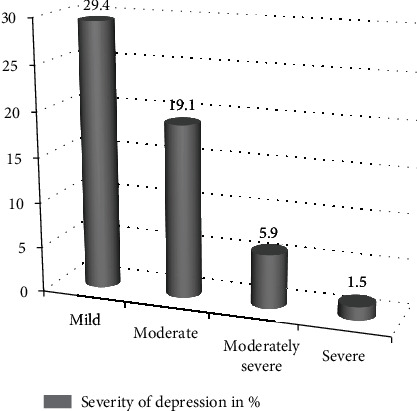
Severity of depression among prisoners in Mekelle General Prison Center, Tigray, Ethiopia, 2019 (*n* = 408).

The prevalence of depression among prisoners in Mekelle General Prison Center was found to be 228 (55.9%; 95% CI: 51.2%, 61%). Of the total number of participants, 120 (29.4%) had mild, 78 (19.1%) had moderate, 24 (5.9%) had moderate to severe, and 6 (1.5%) had severe depression (Figure 4).

### 3.8. Factors Associated with Depression

In bivariate analysis, age, sex, marital status, occupation, residence, occupation, lifetime substance use, history of childhood abuse, history of mental illness, family history of mental illness, having chronic physical illness, subjective report of health status, having a frequent follow-up with a health care provider, suicidal ideation, suicidal attempt, weight loss inside prison, previous incarceration, acceptance of crime, length of charge, type of crime, appropriateness of charge for crime, happy inside prison, thinking the difficulty of life after releasing from prison, work inside prison, satisfaction of day-to-day activities before imprisonment, recreational area in prison, bullying, prison meal, permission to move freely inside prison, and social support were significant at *P* value of 0.2.

Variables with *P* value of <0.2 in bivariate analysis were entered into multivariable analysis to make the association safe. Multivariable logistic regression found that only being unemployed and student (adjusted odds ratio (AOR) = 3.528; 95% confidence interval (CI): 1.041-11.963), lifetime substance use (AOR = 1.943; 95% CI: 1.066-3.541), history of childhood abuse (AOR = 5.147; 95% CI: 1.863-14.224), weight loss in prison (AOR = 4.07; 95% CI: 2.176-7.61), those who were not happy inside prison (AOR = 3.228; 95% CI: 1.76-5.917), subjective response bad quality of meal in prison (AOR = 2.369; 95% CI: 1.165-4.821), length of charge, i.e., sentenced more than six years (AOR = 5.132; CI: 2.176-7.61), poor social support (AOR = 2.087; 95% CI: 1.044-4.172), and moderate social support (AOR = 3.627; 95% CI: 1.725-7.626) were significantly associated with depression (Table 5).

## 4. Discussion

This study tried to assess the prevalence of depression among prisoners. It aimed to identify associated factors of depression among prisoners with specific sociodemographic, health-related, social support, and prison-related factors. This study revealed that the prevalence of depression among prisoners was reported to be 228 (55.9%, 95% CI: 51.2%, 61%). This finding was in line with evidence from Hawassa [30]; the prevalence of depression was rated at 56.4%.

However, this finding was higher than the evidence from general population of Ethiopia (20.5%; 95% CI: 16.5%-24.4%) [19]. The possible explanation for prevalence of depression to be high in prison might be due to stressful environment of the prison, isolation from family, and a guilty feeling about their crime in prison when compared to their counterparts of nonprisoners. The result of this study was higher than the evidence from the study conducted in New York (43%), Iran (42%), Nepal (35.5%), and Nigeria (37%) [23, 24, 27, 28]. The difference could be due to educational status of participants (in New York, 84% of participants had learned at least in high school, while in this study, 42% had learned at least in high school, and in Nigeria, 49% of participants were illiterate, while in this study, only 15% were illiterate), sociodemographic (the study done in New York focuses only on among male prisoners, while in this study, both sexes included) and socioeconomic difference, sample size difference, and tool difference between this study population and the listed studies.

Studies conducted in Jimma and Bahir Dar showed that prevalence of depression was 41.9% and 45.5%, respectively, which was lower than that of the current study result [11, 43]. This difference could be due to tool difference (Beck Depression Inventory (BDI) in Jimma) and sociodemographic (in Jimma, 56.9% were Muslim in religion, while 97% were Christian in this study, and 56.7% were married in Bahir Dar) difference between this study population and the listed studies. On the other hand, this finding is lower than the evidence from the study conducted in India (81%), Pakistan (85%), and Nigeria (72.6%) [25, 26, 31]. The reason for this difference could be tool difference (they used self-reported questionnaire, BDI, and Hamilton rating scale), sample size difference (100 male prisoners only in Pakistan and 252 in Nigeria), sociodemographic (in Pakistan, 100 male prisoners only participated and 78% were married in India), socioeconomic, and cultural difference between this study population and the listed studies.

This study revealed that prisoners who had lifetime substance use were almost two times more likely to develop depression when compared to those who did not use substance in their life (AOR = 1.943; 95% CI: 1.066-3.541). This finding was similar with study results in Hawassa, Jimma, and Pakistan [11, 26, 30]. Other evidences support the association between substance use and depression [6, 44]. In addition, this study showed that history of early childhood abuse was five times more likely to develop depression when compared to those who did not abuse (AOR = 5.147; 95% CI: 1.863-14.224). This finding lined with study result in Pakistan [26]. Prisoners who lost weight in prison were four times more likely to develop depression than those who did not lose weight (AOR = 4.07; 95% CI: 2.176-7.61). This finding is in line with study result in Nepal [27]. Loss of weight is also one symptom of depression [6].

Another reported factor describes that prisoners sentenced more than six years were about five times more likely to develop depression when compared to those who were sentenced lifetime (AOR = 5.13; 95% CI: 1.038-25.378). This finding agreed with study finding in Bahir Dar [43] but disagreed with study result from India [25]. The reason for difference could be prison status (if there is enough and good quality of sleep area, meal, and other daily life needs, those who were sentenced long time can arrange themselves for prison unless they may depressed) and economical difference in India and Ethiopia. The study showed that subjective response of poor prison meal was more than two times more likely to develop depression than those who had response good prison meal (AOR = 2.369; 95% CI: 1.165-4.821). This finding was in line with study conducted in medium security prison in Benin City of Nigeria [31], and other study indicated that food insecurity is significantly associated with depression [45]. Having poor and moderate social support was two times and more than three times more likely to develop depression when compared to those who had strong social support (AOR = 2.087; 95% CI: 1.044-4.172) and (AOR = 3.627; 95% CI: 1.725-7.626), respectively. This finding was similar with study done in Jimma [20] and Southwest Amhara [29]. Lack of social support may lead to increased psychological distress. On the other hand, good social support is vital for those with good health in prevention of depression [46]. Other possible reason could be prisoners are isolated from their family and community. Prisoners who were not happy inside prison were more than three times more likely to be affected by depression when compared to those who were happy prisoners (AOR = 3.228; 95% CI: 1.76-5.917). Being unemployed and student before imprisonment was significantly associated with depression (AOR = 3.528; 95% CI: 1.041-11.963). Other studies did not report these factors which are new associated factors with depression.

## 5. Conclusion

The prevalence of depression among prisoners was high when compared to other studies. Lifetime substance use, being unemployed and student, history of childhood abuse, weight loss inside prison, being sentenced more than six years, those who were not happy inside prison, poor and moderate social support, and quality of prison meal were independent predictors of depression.

## 6. Limitation

When interpreting the findings of the present study, the following limitations should be considered. Being a cross-sectional study cannot show real cause and effect relationship but only temporal relationship. Another possible limitation could be recall bias to lifetime substance use and history of childhood abuse as well as being self-reported prisoners' information could lead to bias.

## 7. Recommendation

It would be better if psychiatry professionals assess depression among prisoners regularly as voluntary service and the prison administrators improve the quality of meal as well as recreational activities inside prison. In addition, improving mental health service in prison and providing interventional study are relevant. Finally, the government should decrease unemployment.

## Figures and Tables

**Table 1:** Sociodemographic characteristic of prisoners in Mekelle General Prison Center, Tigray, Ethiopia, 2019 (*n* = 408).

Variables		Frequency (%)	Depression	
			No, *n* (%)	Yes, *n* (%)
Sex	Male	396 (97.1)	177 (44.7%)	219 (55.3%)
	Female	12 (2.9)	3 (25%)	9 (75%)

Age	18-25	112 (27.5)	60 (53.6%)	52 (46.4%)
	26-33	101 (24.8)	39 (38.6%)	62 (61.4%)
	34-41	105 (25.7)	51 (48.6%)	54 (51.4%)
	≥42	90 (22.1)	30 (33.3%)	60 (66.7%)

Marital status	Single	226 (55.4)	105 (46.5%)	121 (53.5%)
	Married	153 (37.5)	62 (40.3%)	92 (59.7%)
	Separated	21 (5.1)	11 (52.4%)	10 (47.6%)
	Widowed	8 (2)	2 (28.6%)	5 (71.4%)

Religion	Orthodox	393 (96.3)	175 (44.5%)	218 (55.5%)
	Others^a^	15 (3.7)	5 (33.3%)	10 (66.7%)

Residence	Rural	160 (39.2)	82 (51.2%)	78 (48.8)
	Urban	248 (60.8)	98 (39.5%)	150 (60.5%)

Occupation	Farmer	99 (24.3)	52 (52.5%)	47 (47.5%)
	Governmental	45 (11)	23 (51.1%)	22 (48.9%)
	Private work	211 (51.7)	83 (39.3%)	128 (60.7%)
	Student	39 (9.5)	20 (51.3%)	19 (48.7%)
	Others^b^	14 (3.5)	2 (14.3%)	12 (85.7%)

Level of education	Illiterate	62 (15.2)	23 (37.1%)	39 (62.9%)
	Elementary (1-8)	171 (41.9)	83 (48.5%)	88 (51.5%)
	9-12	132 (32.4)	55 (41.7%)	77 (58.3%)
	College and above	43 (10.5)	19 (44.2%)	24 (55.8%)

^a^Muslim, Protestant, and Catholic. ^b^Unemployed and going to Saudi Arabia.

**Table 2:** Clinical/health-related characteristics of prisoners in Mekelle General Prison Center, Tigray, Ethiopia, 2019 (*n* = 408).

Variables		Frequency (%)	Depression	
			No, *n* (%)	Yes, *n* (%)
History of mental illness	Yes	21 (5.1)	5 (23.8%)	16 (76.2%)
	No	387 (94.9)	175 (45.2%)	212 (54.8%)

Family history of mental illness	Yes	35 (8.6)	11 (31.4%)	24 (68.6)
	No	373 (91.4)	169 (45.3%)	204 (54.7%)

Chronic physical illness	Yes	59 (14.5)	18 (30.5%))	41 (69.5%)
	No	349 (85.5)	162 (46.4%)	187 (53.6%)

Subjective response health status	I have good health	330 (80.9)	158 (47.9%)	172 (52.1%)
	I have poor health	78 (19.1)	22 (28.2%)	56 (71.8%)

Frequent follow-up with a health care provider	Yes	69 (16.9)	19 (27.5%)	50 (72.5%)
	No	339 (83.1)	161 (47.5%)	178 (52.5%)

Weight loss inside prison	Yes	229 (56.1)	70 (30.6%)	159 (69.4%)
	No	179 (43.9)	110 (61.5%)	69 (38.5%)

**Table 3:** Prison-related characteristics of prisoners in Mekelle General Prison Center, Tigray, Ethiopia, 2019 (*n* = 408).

Variables		Frequency (%)	Depression	
			No, *n* (%)	Yes, *n* (%)
Previous incarceration	Yes	48 (11.8)	16 (33.3%)	32 (66.7%)
	No	360 (88.2)	164 (45.6%)	196 (54.4%)

Length of charge	<1 year	47 (11.5)	24 (51.1%)	23 (48.9%)
	1-3 years	200 (49)	94 (47%)	106 (53%)
	4-6 years	49 (12)	23 (46.9%)	26 (53.5%)
	>6 years	96 (23.5)	31 (32.3%)	65 (67.7%)
	Lifetime	16 (3.9)	8 (50%)	8 (50%)

Reason for imprisonment	Theft or robbery	151 (37)	71 (47%)	80 (53%)
	Fighting	84 (20.6)	43 (51.2%)	41 (48.8%)
	Rape	29 (7.1)	12 (41.4%)	17 (58.6%)
	Homicide	67 (16.4)	21 (31.3%)	46 (68.7%)
	Corruption	5 (1.2)	3 (60%)	2 (40%)
	Others^e^	72 (17.6)	30 (41.7%)	42 (58.3%)

Acceptance of crime	Yes	236 (57.8)	112 (47.5%)	124 (52.5%)
	No	172 (42.2)	68 (39.5%)	104 (60.5%)

Work inside prison	Yes	290 (71.1)	139 (47.9%)	151 (52.1%)
	No	118 (28.9)	41 (34.7%)	77 (65.3%)

Religious activity inside prison	Always	235 (57.6)	104 (44.3%)	131 (55.7%)
	Sometimes	50 (12.3)	20 (40%)	30 (60%)
	Rare	83 (20.3)	36 (43.4%)	47 (56.6%)
	Never	40 (9.8)	20 (50%)	20 (50%)

Bullying inside prison	Yes	57 (14)	14 (24.6%)	43 (75.4%)
	No	351 (86)	166 (47.3%)	185 (52.7%)

Subjective response for quality of meal inside prison	Good	100 (24.5)	67 (67%)	33 (33%)
	Bad	308 (75.5)	113 (36.7%)	195 (63.3%)

Permission to move freely inside prison	Yes	359 (88)	170 (47.4%)	189 (52.6%)
	No	49 (12)	10 (20.4%)	39 (79.6%)

Happy life before imprisonment	Yes	386 (94.6)	175 (45.3%)	211 (54.7%)
	No	22 (5.4)	5 (22.7%)	17 (77.3%)

Thinking life will be difficult after releasing from prison	Yes	71 (17.4)	19 (26.8%)	52 (73.2%)
	No^d^	337 (82.6)	161 (47.8%)	176 (52.2%)

Happy life inside prison	Yes	142 (34.8)	94 (66.2%)	48 (33.8%)
	No	266 (65.2)	86 (32.3%)	188 (67.7%)

Having recreational area inside prison	Yes	323 (79.2)	155 (48%)	168 (52%)
	No	85 (20.8)	25 (29.4%)	60 (70.6%)

^e^Traffic accident, trying to kill someone, and others. ^d^Lifetime incarceration of prisoners who do not have the chance to release from prison.

**Table 4:** Suicidal characteristics of prisoners in Mekelle General Prison Center, Tigray, Ethiopia, 2019 (*n* = 408).

Variables		Frequency (%)	Depression	
			No, *n* (%)	Yes, *n* (%)
Suicidal ideation	Yes	59 (14.5)	8 (13.6%)	51 (86.4%)
	No	349 (85.5)	172 (49.3%)	177 (50.7%)

Suicidal attempt	Yes	27 (6.6)	3 (11.1%)	24 (88.9%)
	No	381 (93.4)	177 (46.5%)	204 (53.5%)

**Table 5:** Factors associated with depression among prisoners in Mekelle General Prison Center, Tigray, Ethiopia, 2019 (*n* = 408).

Explanatory variables		Depression		Crude odds ratio (COR), 95% CI	AOR, 95% CI	*P* value
		No	Yes			
Occupation	Farmer	52	47	1	1	
	Governmental	23	22	1.058 (0.523-2.142)	0.469 (0.134-1.645)	0.238
	Private work	83	128	**1.706 (1.054-2.762)**	2.606 (0.982-6.919)	0.054
	Others^a^	22	31	1.559 (0.795-3.059)	**3.528 (1.041-11.96)**	**0.043** ^∗∗^

Lifetime substance use	Yes	55	117	**2.396 (1.590-3.609)**	**1.943 (1.066-3.541)**	**0.030** ^∗∗^
	No	125	111	1	1	

History of childhood abuse	Yes	9	44	**4.543 (2.153-9.586)**	**5.147 (1.86-14.224)**	**0.002** ^∗∗^
	No	171	184	1	1	

Weight loss	Yes	70	159	**3.621 (2.399-5.467)**	**4.07 (2.176-7.61)**	**0.000** ^∗∗^
	No	110	69	1	1	

Length of charge	Less than one year	24	23	0.958 (0.308-2.981)	4.21 (0.57-31.12)	0.159
	1-3 years	94	106	1.128 (0.487-3.123)	3.727 (0.567-24.50)	0.171
	4-6 years	23	26	1.13 (0.365-3.497)	4.717 (0.681-32.66)	0.116
	More than 6 years	31	65	**2.097 (0.72-6.109)**	**5.13 (1.038-25.378)**	**0.045** ^∗∗^
	Lifetime	8	8	1	1	

Happy life inside prison	Yes	94	48	1	1	
	No	86	180	**4.099 (2.660-6.316)**	**3.228 (1.76-5.917)**	**0.000** ^∗∗^

Prison meal	Good	67	33	1	1	
	Bad	113	195	**3.504 (2.175-5.645)**	**2.37 (1.165-4.821)**	**0.017** ^∗∗^

Social support	Poor social support	56	100	**2.262 (1.413-3.622)**	**2.087 (1.044-4.172)**	**0.037** ^∗∗^
	Moderate social support	48	68	**1.794 (1.087-2.962)**	**3.627 (1.725-7.626)**	**0.001** ^∗∗^
	Strong social support	76	60	1	1	

^∗∗^Significant at *P* value < 0.05. ^a^Students, unemployed, and those going to Saudi.

## Data Availability

The datasets used and/or analyzed during the current study are available from the corresponding author on reasonable request.

## Abbreviations

ACSH: Ayder Comprehensive Specialized Hospital
AOR: Adjusted odds ratio
BDI: Beck Depression Inventory
CI: Confidence interval
ICCMH: Integrated Clinical and Community Mental Health
LMICs: Low- and middle-income countries
OR: Odds ratio
OSSS: Oslo Social Support Scale
PHQ: Patient Health Questionnaire
SPSS: Statistical Package for Social Sciences
WHO: World Health Organization.

## References

[1] Fazel S., Hayes A. J., Bartellas K., Clerici M., Trestman R. (2016). Mental health of prisoners: prevalence, adverse outcomes, and interventions. *The Lancet Psychiatry*.

[2] Ali Y., Yigzaw N. (2016). Prevalence of common mental disorders and associated factors among prisoners in Debre Markos Town Correctional Institution, North-West, Ethiopia. *International Journal of Mental Health & Psychiatry*.

[3] Mundt A. P., Alvarado R., Fritsch R. (2013). Prevalence rates of mental disorders in Chilean prisons. *PLoS One*.

[4] Forrester A., Slade K. (2014). Preventing self-harm and suicide in prisoners: job half done. *The Lancet.*.

[5] Birhaner D., Ispas M. *Ethiopia has the second highest number of prisoners in Africa*.

[6] DSM-5 American Psychiatric Association (2013). *Diagnostic and statistical manual of mental disorders*.

[7] World Health Organization (2017). *Depression and other common mental disorders*.

[8] Cramer V. (2014). *The Prevalence of Mental Disorders among Convicted Inmates in Norwegian Prisons*.

[9] Dachew B. A., Fekadu A., Kisi T., Yigzaw N., Bisetegn T. A. (2015). Psychological distress and associated factors among prisoners in North West Ethiopia: cross-sectional study. *International Journal of Mental Health Systems*.

[10] Mweene M. T., Siziya S. (2016). Prevalence of mental illness among inmates at Mukobeko maximum security prison in Zambia: a cross-sectional study. *Journal of Mental Health and Human Behavior*.

[11] Abdu Z., Kabeta T., Dube L., Tessema W., Abera M. (2018). Prevalence and associated factors of depression among prisoners in Jimma Town Prison, South West Ethiopia. *Psychiatry Journal*.

[12] Prins S. J. (2014). Prevalence of mental illnesses in US state prisons: a systematic review. *Psychiatric Services*.

[13] Lazarus R., Freeman M. (2009). Primary-level mental health care for common mental disorder in resource-poor settings: models & practice. *A literature review*.

[14] World Health Organization (2001). Mental disorders affect one in four people.

[15] Patel V., Prince M. (2010). Global mental health. *JAMA*.

[16] Thyloth M., Singh H., Subramanian V. (2016). Increasing burden of mental illnesses across the globe: current status. *Indian Journal of Social Psychiatry*.

[17] Prince M., Patel V., Saxena S. (2007). No health without mental health. *The Lancet*.

[18] International Monetary Fund (2012). The Federal Democratic Republic of Ethiopia: Staff Report for the 2012 Article IV Consultation. *IMF Staff Country Reports*.

[19] Bifftu B. B., Takele W. W., Guracho Y. D., Yehualashet F. A. (2018). Depression and its help seeking Behaviors: a systematic review and meta-analysis of community survey in Ethiopia. *Depression Research and Treatment*.

[20] Wang J., Wu X., Lai W. (2017). Prevalence of depression and depressive symptoms among outpatients: a systematic review and meta-analysis. *BMJ Open*.

[21] Fazel S., Danesh J. (2002). Serious mental disorder in 23 000 prisoners: a systematic review of 62 surveys. *The Lancet*.

[22] Fazel S., Seewald K. (2012). Severe mental illness in 33 588 prisoners worldwide: systematic review and meta-regression analysis. *The British Journal of Psychiatry*.

[23] Rowell T. L., Draine J., Wu E. (2011). Depression in a random sample of incarcerated African-American men. *Psychiatric Services*.

[24] Valizadeh R., Veisani Y., Delpisheh A., Kikhavani S., Sohrabnejad A. (2017). Major depression and psychiatric disorders in Iranian prisoners based on a clinical interview: a systematic review and meta-analysis. *Shiraz E-Medical Journal*.

[25] Datta P. V., Vijaya M. N., Krishna I. V., Bai B. S., Sharon M. T., Ramam S. (2015). Prevalence of depression and assessment of its severity among prisoners of central prison, Rajahmundry, India. *Indo American Journal of Pharmaceutical Research*.

[26] Shahid I., Aftab M. A., Yousaf Z., Naqvi S. H., Hashmi A. M. (2014). Prevalence of depression among male prisoners at an urban jail in Pakistan. *HealthMED*.

[27] Shrestha G., Yadav D. K., Sapkota N. (2017). Depression among inmates in a regional prison of eastern Nepal: a cross-sectional study. *BMC Psychiatry*.

[28] Valizadeh R., Veisani Y., Delpisheh A., Kikhavani S., Sohrabnejad A. (2017). Major depression and psychiatric disorders in Iranian prisoners based on a clinical interview: A systematic review and meta-analysis. *Shiraz E-Medical Journal*.

[29] Beyen T. K., Dadi A. F., Dachew B. A., Muluneh N. Y., Bisetegn T. A. (2017). More than eight in every nineteen inmates were living with depression at prisons of Northwest Amhara Regional State, Ethiopia, a cross sectional study design. *BMC Psychiatry*.

[30] Bedaso A., Kediro G., Yeneabat T. (2018). Factors associated with depression among prisoners in southern Ethiopia: a cross-sectional study. *BMC Research Notes*.

[31] Osasona S. O., Koleoso O. N. (2015). Prevalence and correlates of depression and anxiety disorder in a sample of inmates in a Nigerian prison. *The International Journal of Psychiatry in Medicine*.

[32] Nwaopara U., Stanley P. (2015). Prevalence of depression in Port Harcourt prison. *Journal of Psychiatry*.

[33] Larney S., Topp L., Indig D., O'Driscoll C., Greenberg D. (2012). A cross-sectional survey of prevalence and correlates of suicidal ideation and suicide attempts among prisoners in New South Wales, Australia. *BMC Public Health*.

[34] Cunniffe C., Van de Kerckhove R., Williams K., Hopkins K. (2012). Estimating the prevalence of disability amongst prisoners: results from the Surveying Prisoner Crime Reduction (SPCR) survey. *Ministry of Justice*.

[35] Moussavi S., Chatterji S., Verdes E., Tandon A., Patel V., Ustun B. (2007). Depression, chronic diseases, and decrements in health: results from the World Health Surveys. *The Lancet*.

[36] Evans-Lacko S., Koeser L., Knapp M., Longhitano C., Zohar J., Kuhn K. (2016). Evaluating the economic impact of screening and treatment for depression in the workplace. *European Neuropsychopharmacology*.

[37] Majekodunmi O. E., Obadeji A., Oluwole L. O., Oyelami R. O. (2017). Depression in prison population: demographic and clinical predictors. *Journal of Forensic Science and Medicine*.

[38] Dadi A. F., Dachew B. A., Kisi T., Yigzaw N., Azale T. (2016). Anxiety and associated factors among prisoners in North West of Amhara Regional State, Ethiopia. *BMC Psychiatry*.

[39] Fazel S., Danesh J. (2002). Serious mental disorder in 23,000 prisoners: a systematic review of 62 surveys. *Lancet*.

[40] Abraha H., Hadish G., Aligaz B., Eyas G., Workelule K. (2018). Antimicrobial resistance profile of Staphylococcus aureus isolated from raw cow milk and fresh fruit juice in Mekelle, Tigray, Ethiopia. *Journal of Veterinary Medicine and Animal Health*.

[41] Abiola T., Udofia O., Zakari M. (2013). Psychometric properties of the 3-item Oslo Social Support Scale among clinical students of Bayero University Kano, Nigeria. *Malaysian Journal of Psychiatry*.

[42] Kroenke K., Spitzer R. L., Williams J. B. (2001). The PHQ-9. *Journal of General Internal Medicine*.

[43] Alemayehu F., Ambaw F., Gutema H. (2019). Depression and associated factors among prisoners in Bahir Dar Prison, Ethiopia. *BMC Psychiatry*.

[44] Chirita V., Untu I. (2016). Kaplan and Sadock’s synopsis of psychiatry: behavioural sciences/clinical psychiatry. *Bulletin of Integrative Psychiatry*.

[45] Palar K., Kushel M., Frongillo E. A. (2015). Food insecurity is longitudinally associated with depressive symptoms among homeless and marginally-housed individuals living with HIV. *AIDS and Behavior*.

[46] Duko B., Gebeyehu A., Ayano G. (2015). Prevalence and correlates of depression and anxiety among patients with tuberculosis at WolaitaSodo University Hospital and Sodo Health Center, WolaitaSodo, South Ethiopia, cross sectional study. *BMC Psychiatry*.

